# Identification of PD‐1‐Related Genes as Prognostic Biomarkers in Lung Adenocarcinoma

**DOI:** 10.1155/humu/8948469

**Published:** 2026-08-25

**Authors:** Bin Jia, Ting Gong, Chen Chen, Bingsheng Sun, Zhenfa Zhang

**Affiliations:** ^1^ Lung Cancer Department, Tianjin Medical University Cancer Institute and Hospital National Clinical Research Center for Cancer, Key Laboratory of Cancer Prevention and Therapy, Tianjin′s Clinical Research Center for Cancer, Tianjin, China, tmucih.com; ^2^ Department of Oncology, Tianjin Medical University General Hospital, Tianjin, China, tjmugh.com.cn

**Keywords:** drug sensitivity, immune cell infiltration, lung adenocarcinoma, migration and invasion, *PD-1*, prognostic model

## Abstract

**Background:**

The PD‐1/PD‐L1 axis plays a critical role in suppressing T‐cell activation and facilitating immune evasion in lung adenocarcinoma (LUAD). This study focused on PD‐1‐related genes to develop a robust prognostic prediction model for LUAD.

**Methods:**

Immune scores were calculated using the ESTIMATE algorithm, and immune‐related gene modules were identified through WGCNA. Differentially expressed genes (DEGs) were identified between normal and tumor tissues, and between high and low PD‐1 expression groups, using the limma package. Univariate and multivariate Cox regression analyses were performed to construct a prognostic risk model, which was subsequently evaluated using time‐dependent ROC curve analysis with the timeROC package. Biomarker expression and function were validated in LUAD cells via western blot (WB), CCK‐8, wound healing, and Transwell assays. Functional enrichment analysis was conducted using the clusterProfiler package. The tumor microenvironment (TME) was characterized by integrating MCPcounter, ESTIMATE, and ssGSEA. Drug sensitivity was predicted using the pRRophetic R package.

**Results:**

WGCNA showed that genes in the brown module were significantly positively correlated with the immune score, and these genes were predominantly enriched in biological processes such as neutrophil activation and cytokine activity. By intersecting the brown module genes, DEGs, and PD‐1‐related genes, we constructed a risk prognosis model comprising nine key genes (*ADA2*, *CD53*, *HLA-DMA*, *ITGAX*, *MYO1F*, *NCKAP1L*, *PTPRC*, *RENBP*, and *TNFAIP8L2*). The model demonstrated robust predictive performance, with high AUC values in time‐dependent ROC analysis. Patients within the high‐risk group exhibited a worse prognosis. Additionally, RiskScore for patients in the high‐risk group was closely associated with immune infiltration, immune checkpoint expression, and drug sensitivity. The low‐risk group exhibited higher levels of immune cell infiltration, including T cells, CD8^+^ T cells, and B lineage cells. Furthermore, in vitro experiments have demonstrated that silencing the representative gene *MYO1F* significantly inhibited the migration and invasion of LUAD cells, whereas *PTPRC* knockdown promoted these capacities. Drug sensitivity analysis identified distinct candidate therapeutic agents for the high‐ and low‐risk groups.

**Conclusion:**

In conclusion, this study developed a robust prognostic prediction model for LUAD, contributing to personalized therapeutic strategies.

## 1. Introduction

Lung adenocarcinoma (LUAD) is the most frequent histological subtype among non–small cell lung cancer (NSCLC) types, accounting for approximately 40% of all malignancies [1–3]. In 2023, lung cancer caused an estimated 2.04 million deaths worldwide, remaining the leading cause of cancer‐related mortality, and global cancer deaths are projected to reach 18.6 million by 2050 [4]. The prognosis for LUAD patients remains poor, with a 5‐year survival rate below 20% [5, 6]. Prolonged tobacco exposure is the leading etiological factor [7]. Most patients are diagnosed at an advanced stage and typically receive conventional chemotherapy and radiotherapy, which are associated with limited efficacy and significant toxicity [8]. In contrast, targeted therapies act specifically on cancer cells with minimal effects on normal cells [9], suggesting that continuous exploration for new therapeutic targets is of crucial significance to further improve the treatment and the prognosis of LUAD patients [10, 11].

PD‐1 is a pivotal immune checkpoint regulator that exerts its effects by suppressing both the innate and adaptive immune responses [12]. Specifically, PD‐1 inhibits the activation and proliferation of T cells and the production of cytokines [13]. Whereas increased tumor‐infiltrating T lymphocytes (TILs) are generally correlated with favorable clinical outcomes, cancer cells take advantage of the PD‐1 signaling pathway to escape immune surveillance, which represents a characteristic feature of malignant progression [14]. Mechanistically, tumor cells overexpress PD‐L1, which binds to PD‐1 on T cells, inducing immune exhaustion and acquired resistance [15]. The PD‐1/PD‐L1 axis has been clinically validated as a predictive biomarker in multiple malignancies [16]. In LUAD, PD‐L1 immunohistochemical (IHC) staining of pathological specimens directly guides therapeutic decision‐making [17]. ICIs targeting PD‐1 or PD‐L1 have significantly transformed LUAD treatment by rejuvenating exhausted T cells and re‐establishing antitumor immunity [18]. Emerging evidence further supports their synergistic potential when combined with other modalities, such as antiangiogenic therapies, to overcome TME‐mediated immunosuppression [19]. Consequently, establishing a prognostic model based on PD‐1‐related genes is urgently needed to guide individualized treatment for patients with LUAD [20].

In this study, we constructed and validated a multigene prognostic model for LUAD based on PD‐1‐related genes and systematically explored its association with the tumor immune microenvironment and drug sensitivity. By integrating multidimensional immune information, this study provides novel molecular evidence for personalized prognostic assessment and optimized immunotherapy strategies in LUAD.

## 2. Material and Methods

### 2.1. Data Gathering and Its Subsequent Processing

Gene expression data and clinical information of patients with LUAD were sourced from The Cancer Genome Atlas (TCGA) (https://portal.gdc.cancer.gov) [21] database as a training set. After excluding samples with missing survival data, 500 tumor and 59 normal samples were retained. RNA‐seq data were converted to FPKM format and log_2_‐transformed. Microarray datasets GSE30219 and GSE3141 were downloaded from the Gene Expression Omnibus (GEO) as independent validation sets. Based on the annotation file, probes were transformed into gene symbols. After excluding normal tissue samples and those without clinical follow‐up information or overall survival (OS) data, 83 and 111 LUAD samples from the GSE30219 and GSE3141 datasets were retained for further analysis, respectively.

### 2.2. Immunological Signature Score Analysis

The immunological signature scores were calculated using ESTIMATE [22]. In the TCGA cohort, patients were divided into PDCD1 high‐expression and low‐expression groups according to the median PDCD1 expression level. The limma package was used for differential expression analysis, with the screening thresholds set as |log2FC| > log2(1.5) and adj.*p* < 0.05.

### 2.3. WGCNA

WGCNA was performed using the WGCNA package [23] to identify gene modules that were related to the ESTIMATE score. The soft threshold *β* was determined using the pickSoftThreshold function to ensure a scale‐free network topology. Hierarchical clustering was then conducted with minModuleSize = 60. Based on module–trait correlation analysis, genes in the modules closely related to the immune scores were obtained for subsequent analysis.

### 2.4. Enrichment Analysis

To analyze the genes within the selected module, the R package clusterProfiler [24] was employed to perform GO and KEGG analyses.

### 2.5. Construction and Validation of a Risk Model

Using the limma package [25], we identified DEGs between normal and tumor tissues under the screening criteria of |log2(FC)| > log2(1.5) and *p* < 0.05. The moderated *t*‐test based on empirical Bayesian correction was used to calculate the differential expression of genes, and the Benjamini–Hochberg method was applied for multiple testing correction to obtain the false discovery rate (FDR). Subsequently, to reduce candidate genes within the risk model, we performed univariate and multivariate Cox regression analysis using the survival package (Version 3.2‐7) [26].

Based on the obtained biomarkers, the risk model was formulated as follows: RiskScore = *Σ* *β* *i* × Expi, where *i* is the index of each gene and *β* is the Cox regression coefficient of the gene. Based on the median RiskScore, patients were stratified into high‐risk and low‐risk groups. Survival differences were assessed by Kaplan–Meier (KM) survival analysis, and the model performance was evaluated according to the receiver operating characteristic (ROC) curve at 1, 3, and 5 years using the timeROC R package [27]. This study constructed and validated the prognostic model with OS as the primary endpoint. Disease‐specific survival (DSS) served as a supplementary endpoint for robustness analysis only in the TCGA cohort. Specifically, DSS was defined as the time to tumor‐related death, whereas non–tumor‐related death and surviving patients were regarded as censored data.

The protein expression of the prognostic model genes was further validated using public proteomic databases. Specifically, based on the CPTAC‐LUAD proteomic data provided by the UALCAN platform (https://ualcan.path.uab.edu/), we analyzed the differential protein expression of key genes between LUAD tumor tissues and normal tissues.

### 2.6. Analysis of the Tumor Immune Microenvironment

The MCPcounter package [28] was used to calculate the scores of 10 types of immune cells. The ESTIMATE algorithm [22] was used to assess the infiltration score of the overall immune TME. The ssGSEA function in the GSVA [29] package was used to compute the scores of 28 distinct types of immune cells [30].

### 2.7. Drug Sensitivity Analysis

The pRRophetic package [31] was used to predict the sensitivity of patients in the TCGA‐LUAD cohort to commonly used chemotherapeutic drugs. The correlation between the IC_50_ values of these drugs and the RiskScores was analyzed.

### 2.8. Cell Lines and Transfection

LUAD cell lines H2228 (BNCC340351) and A549 (BNCC337696) were purchased from Beina Biotechnology Co. Ltd., Xinyang, China. Human normal bronchial epithelial cell line BEAS‐2B (C5382, BDBio Co. Ltd., Hangzhou, China) was also obtained. All cell lines were authenticated by short tandem repeat analysis. H2228 cells, BEAS‐2B cells, and A549 cells were cultured in RPMI‐1640 medium (BNCC338360), Dulbecco′s Modified Eagle Medium (C5382‐500), and F‐12K medium (BNCC338550), respectively, each supplemented with 10% FBS. All the cells were cultured at 37°C in an environment containing 5% CO_2_.

For silencing the myosin 1F (*MYO1F*) expression in LUAD cells, the small interfering RNAs of *MYO1F* (si‐*MYO1F*#1: 5 ^′^ AGCAAATCTTTATCGAACTTACC 3 ^′^ and si‐*MYO1F*#2: 5 ^′^ CAGCATCAATCGGAACTTCGTCG 3 ^′^) and si‐NC were ordered from RiboBio (Guangzhou, China). Following the manufacturer′s manual, LUAD cell lines (H2228 and A549) were transfected with siRNA using Lipofectamine 2000 Transfection Reagent (11668019, Invitrogen, Carlsbad, California, United States). The protein tyrosine phosphatase receptor Type C (*PTPRC*) overexpression plasmid was constructed using the pcDNA3.1 vector (V790‐20, Invitrogen, Carlsbad, California) by RiboBio Co. Ltd. (Guangzhou, China). Following the protocol, the constructed overexpression plasmid was transfected into oe‐*PTPRC* cells using Lipofectamine 2000 Reagent (Invitrogen) for a duration of 48 h.

### 2.9. Quantitative Reverse Transcription PCR (qRT‐PCR)

Total RNA of cells was extracted employing TRIzol reagent (R0016, Beyotime, Shanghai, China) and reverse‐transcribed into cDNA using BeyoRT II First Strand cDNA Synthesis Kit (D7168M, Beyotime, China). Thereafter, qRT‐PCR amplification was conducted using BeyoFast SYBR Green qPCR Mix (D7260, Beyotime, China) on a CG‐05 PCR System (Heal Force, Shanghai, China). See Table 1 for the primers used. The expression levels of genes were calculated using the 2^−*ΔΔ*CT^ method, with *GAPDH* as an internal reference gene.

**Table 1:** Primer sequences utilized in this study.

Gene	Forward primer	Reverse primer
*ADA2*	5 ^′^ CAGGAAGTAGCTCAGAAGTT 3 ^′^	5 ^′^ CATCTCTTCTTCCAGATTTC 3 ^′^
*CD53*	5 ^′^ GCTATGCGAAAGCAAGACTGTGG 3 ^′^	5 ^′^ GTCAATCTGGCAGTTCAGGGTC 3 ^′^
*HLA_DMA*	5 ^′^ GTTCTGCGAGTGGATGATCCAG 3 ^′^	5 ^′^ TGTTGGGCTTGCCAAACTCCAG 3 ^′^
*ITGAX*	5 ^′^ GATGCTCAGAGATACTTCACGGC 3 ^′^	5 ^′^ CCACACCATCACTTCTGCGTTC 3 ^′^
*MYO1F*	5 ^′^ CTTTGCCCGAACCATCCAGAAG 3 ^′^	5 ^′^ CCGACGAAGTTCCGATTGATGC 3 ^′^
*NCKAP1L*	5 ^′^ TCTGTGCTGAGCAGCGAAACCT 3 ^′^	5 ^′^ TGACTCTCAGCTCCTGGCTTGT 3 ^′^
*PTPRC*	5 ^′^ CTTCAGTGGTCCCATTGTGGTG 3 ^′^	5 ^′^ CCACTTTGTTCTCGGCTTCCAG 3 ^′^
*RENBP*	5 ^′^ CCATGATGCTACTGAACCTGGTG 3 ^′^	5 ^′^ CCTCTGACACATTCTCCAGCAC 3 ^′^
*TNFAIP8L2*	5 ^′^ GCTGCTAGAGTTGGTGGAACAC 3 ^′^	5 ^′^ CTTGCCAAGGTGCTGAGTGAAG 3 ^′^
*GAPDH*	5 ^′^ CTGGGCTACACTGAGCACC 3 ^′^	5 ^′^ AAGTGGTCGTTGAGGGCAATG 3 ^′^

### 2.10. Cell Viability Experiment

The cell viability of H2228 and A549 cells transfected with si‐*MYO1F* and oe‐*PTPRC* was measured by the CCK‐8 assay. In brief, the transfected cells were added to a 96‐well plate at a density of 5 × 10^3^ cells/well. Then, 10 *μ*L CCK‐8 reagent (C0038, Beyotime, China) was added to each well at 24, 48, and 72 h and incubated for 30 min. Finally, the absorbance value (OD 450 nm) was measured with a microplate reader (Synergy Neo2, Agilent, Santa Clara, California, United States).

### 2.11. Cell Migration and Invasion Assays

Cell migration of *MYO1F*‐silenced and *PTPRC-*overexpressed H2228 and A549 cells was assessed by wound healing assay. Specifically, the transfected cells (5 × 10^4^ cells/well) were placed into a 6‐well plate containing appropriate complete medium at 37°C with 5% CO_2_. When a monolayer was formed, an artificial wound was created using a 100 *μ*L pipette tip. Subsequently, the cells were rinsed twice in phosphate‐buffered saline (PBS) and observed under an inverted microscope (CKX53, Olympus, Tokyo, Japan) to assess their migratory capacity.

Additionally, the cell invasion of *MYO1F*‐silenced and *PTPRC*‐overexpressed H2228 and A549 cells was measured by a Transwell assay. Briefly, the transfected cells were seeded into the upper Transwell chamber precoated with Matrigel. Meanwhile, the lower Transwell chamber contained complete culture medium. After incubation for 48 h, the invaded cells were fixed with 4% paraformaldehyde, stained with 0.1% crystal violet, and observed under the same microscope as above.

### 2.12. Western Blot (WB) Assay

WB analysis was performed to detect the protein expression levels of nine key genes and to verify the efficiency of *MYO1F* knockout and *PTPRC* overexpression. Total cellular protein was extracted using RIPA lysis buffer (Cat. No. 89901, Thermo Fisher Scientific, Waltham, Massachusetts, United States). The protein concentration was quantified with a BCA protein assay kit (Cat. No. GK10009, GlpBio, Montclair, California, United States) to standardize the loading amount. After protein extraction, samples were separated by SDS‐PAGE and transferred onto PVDF membranes (Cat. No. FFP32, Beyotime, Shanghai, China). The membranes were blocked with 5% skim milk for 2 h to eliminate nonspecific binding. Detailed information on all primary antibodies used in this study is listed in Supporting Information 3: Table S1. The membranes were incubated with primary antibodies overnight at 4°C, followed by incubation with goat antirabbit IgG H&L (HRP) secondary antibody (dilution ratio, 1:2500; Cat. No. ab205718, Abcam, Cambridge, United Kingdom) for 1.5 h at room temperature. Protein bands were visualized using BeyoECL Plus chemiluminescence reagent (Cat. No. P0018S, Beyotime Biotechnology). The grayscale values of target bands were subsequently quantified and analyzed via ImageJ software (Version 1.8.0).

### 2.13. Statistical Tests

Data analysis was performed using R (Version 3.6.0) and GraphPad Prism (Version 8.0). For continuous variables, the Wilcoxon rank‐sum test was utilized to determine differences between two groups, and correlations were evaluated by Spearman′s rank correlation analysis. Survival differences among groups were compared using the log‐rank test. Welch′s *t*‐test was used for two‐group comparisons, whereas one or two‐way ANOVA was applied for three or more groups. All in vitro experiments were performed in triplicate. *p* < 0.05 was considered statistically significant.

## 3. Results

### 3.1. Immunological Characteristics in LUAD

The estimate score, stromal score, and immune score in the tumor group were notably lower than in the adjacent nontumor tissues (Figure 1A, *p* < 0.0001). Patients were stratified into high and low immune score groups based on the median estimate score. It was found that patients with a high immune score demonstrated a more favorable prognosis (Figure 1B, *p* < 0.05). Additionally, PD‐1 expression was notably higher in the tumor group (Figure 1C, *p* < 0.0001). Differential analysis between the high‐ and low‐expression groups identified 395 upregulated genes and 30 downregulated genes (Figure 1D; Supporting Information 4: Table S2). Further comparison revealed that PD‐1 expression was significantly higher in the low‐risk group than in the high‐risk group (Figure 1E, *p* < 0.0001).

Figure 1Expressions of immune‐correlated parameters in the normal and tumor groups. (A) The expression of the immune score in normal and tumor groups ( ^∗∗∗∗^ *p* < 0.0001). (B) The prognosis for the groups with high and low immune scores. (C) PD‐1 gene level in normal and tumor groups. (D) Volcano plot showing the distribution of differentially expressed genes between PDCD1 high‐ and low‐expression groups stratified by the median PDCD1 expression in the TCGA cohort. (E) Comparison of PD‐1 expression between high‐ and low‐risk groups, with significantly elevated PD‐1 levels in the low‐risk group ( ^∗∗∗∗^ *p* < 0.0001).

**Figure:**
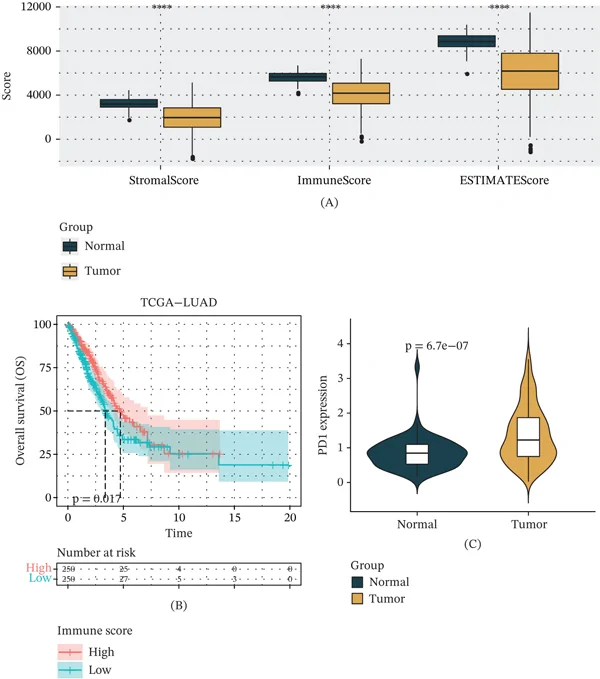
(A)

**Figure:**
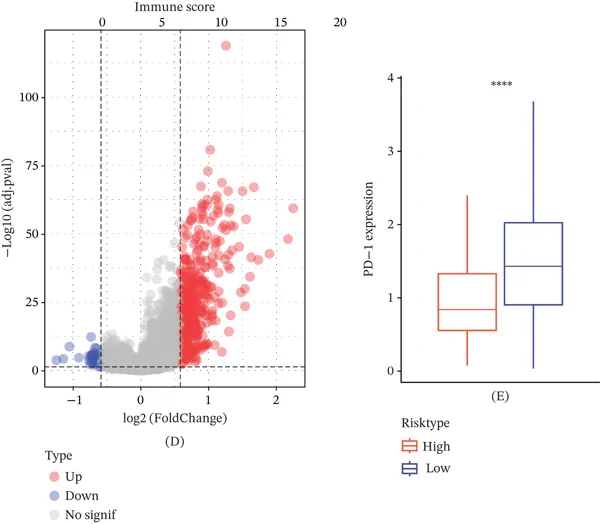
(B)

### 3.2. Employing WGCNA to Identify Immune‐Associated Genes

**Figure 2:**
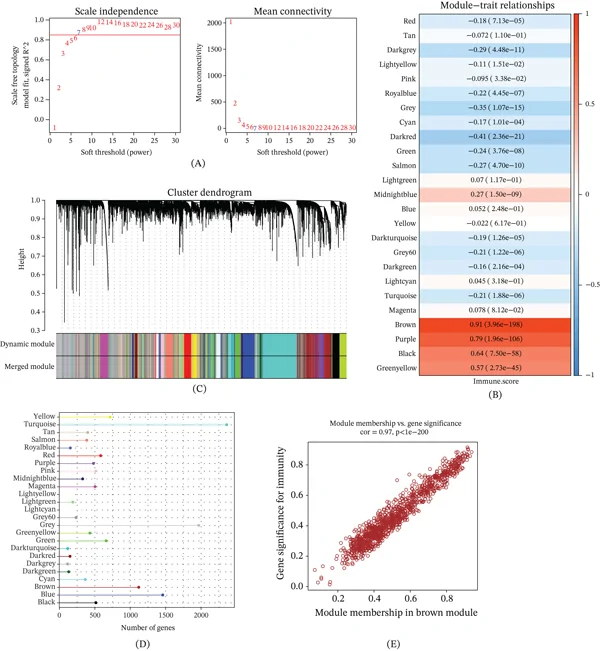
Results of WGCNA. (A) Scale‐free fit index and average connectivity across various soft‐threshold powers (*β*). (B) A gene dendrogram generated through clustering using 1‐TOM. (C) The connection between the module eigengenes of every module and the traits. (D) Gene number in every module. (E) The association between the module membership of genes within the brown module and the immune scores. Each point represents an individual gene. The *x*‐axis depicts the degree of a gene′s membership in the brown module (i.e., the extent of the relation between the gene and the brown module), and the *y*‐axis shows the association between the gene and the immune score.

WGCNA was conducted on the samples to cluster and screen coexpression modules. When the soft‐thresholding power *β* was 7, the scale‐free topology fit index first approached 0.90, indicating that the network was basically scale‐free. Meanwhile, the mean connectivity did not decrease significantly; therefore, *β* = 7 was determined as the optimal soft threshold (Figure 2A). Subsequently, hierarchical clustering was used for classifying gene modules. After merging the modules, 25 coexpression modules were clustered (Figure 2B). Further analysis was conducted to analyze the association between each module and immune score, with the brown module showing a strong correlation with the immune score (Figure 2C). The brown module contained 1114 genes (Figure 2D), and the gene membership was significantly positively correlated with the immune score (cor = 0.97, *p* = 1e − 200) (Figure 2E).

### 3.3. Enrichment Analysis

The KEGG enrichment results showed significant enrichment in pathways such as tuberculosis, osteoclast differentiation, Influenza A, phagosome, hematopoietic cell lineage, and lysosome (Figure 3A). GO analysis revealed enrichment of the brown module genes in biological processes including regulation of neutrophil‐mediated immunity, leukocyte activation, neutrophil activation, and neutrophil degranulation involved in immune response (Figure 3B). These genes were enriched in the cellular component terms including secretory granule membrane, endosome membrane, vacuolar membrane, side of membrane, lysosomal membrane, tertiary granule, and specific granule (Figure 3C). In molecular function terms, these genes were significantly enriched in pathways including carbohydrate binding, cytokine activity, cytokine receptor binding, and cytokine receptor activity (Figure 3D).

Figure 3Results of enrichment analysis. (A) KEGG analysis on the brown module genes. (B) The brown module genes were subjected to GO analysis in the BO term. (C) The brown module genes were subjected to GO analysis in the CC term. (D) The brown module genes were subjected to GO analysis in the MF term.

**Figure:**
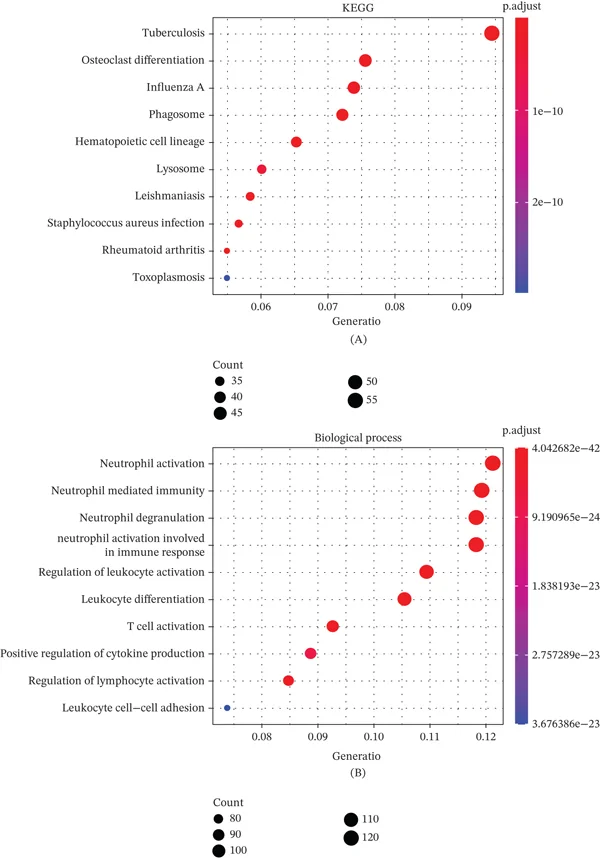
(A)

**Figure:**
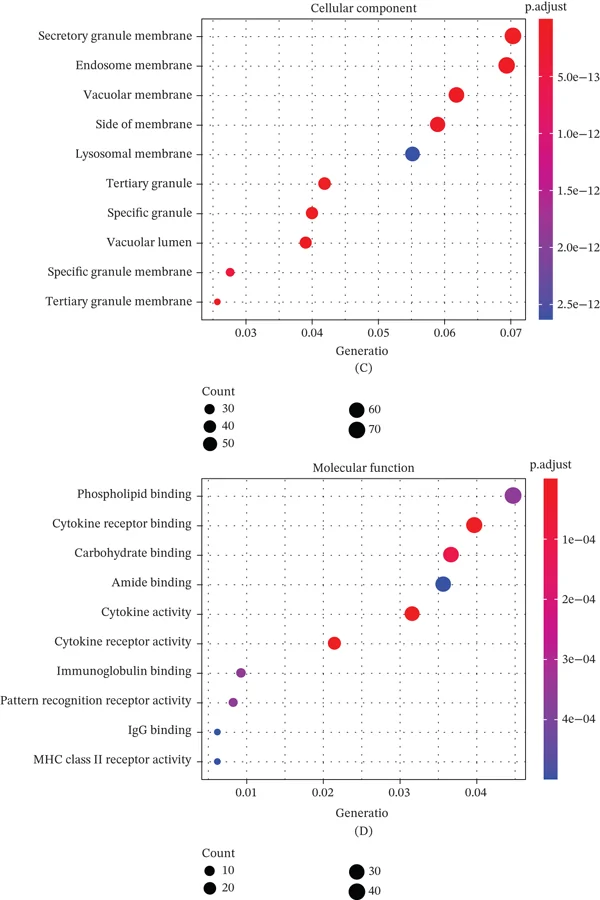
(B)

### 3.4. Development and Validation of a Risk Model

A total of 1959 downregulated DEGs and 2290 upregulated DEGs were identified between the normal and tumor tissue samples in the LUAD‐TCGA database (Figure 4A). Subsequently, 395 upregulated DEGs and 30 downregulated DEGs were identified between the low‐ and high‐expression groups of PD‐1. Next, a total of 96 overlapping genes were identified from the intersection of the brown module genes, the DEGs between normal and tumor tissues, and those between the two expression groups of PD‐1 (Figure 4B). Univariate Cox regression analysis demonstrated that 34 of these DEGs were significantly related to the prognosis of LUAD (*p* < 0.05, Supporting Information 5: Table S3). Multivariate Cox regression analysis was implemented, and finally, we obtained nine independent prognostic genes for constructing a RiskScore model (Figure 4C):

RiskScore=−0.27220.70953×ADA+×CD+−0.204×HLA_DMA+−0.417×ITGAX+0.56810.8811×MYOF+×NCKAPL+−1.033×PTPRC+−0.161×RENBP+−0.393×TNFAIP82L.

Using the formula, patients were assigned a prognosis‐related RiskScore and then divided into high‐ and low‐risk groups by the optimal cutoff value of RiskScore.

Figure 4Development and verification of the risk model. (A) Volcano plot depicting the DEGs in tumor and normal groups within the TCGA dataset. (B) Venn diagram showing the overlap among the DEGs between the low and high PD‐1 expression groups, those between normal and tumor groups, and genes from the brown module. Venn diagram of the intersection of the DEGs between the normal and tumor groups, the DEGs between the low and high PD‐1 expression groups, and the brown module genes. (C) Risk coefficients of the key genes in the training set. (D) ROC curve of the TCGA training set. (E) KM curve of the DSS in the TCGA training set. (F) KM curve of the OS in the TCGA training set. (G) Distribution of survival status between high‐risk and low‐risk groups stratified by the median cutoff value of RiskScore in the TCGA dataset. (H) Distribution of survival status in high‐risk and low‐risk groups stratified by the median cutoff of risk score in the GSE30219 dataset. (I) ROC curve of the GSE30219 validation set. (J) KM curve of OS in the GSE30219 validation set.

**Figure:**
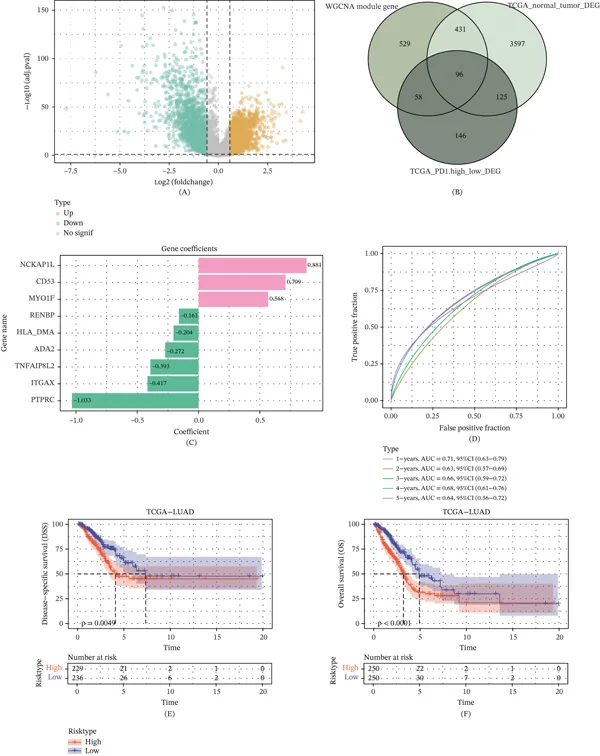
(A)

**Figure:**
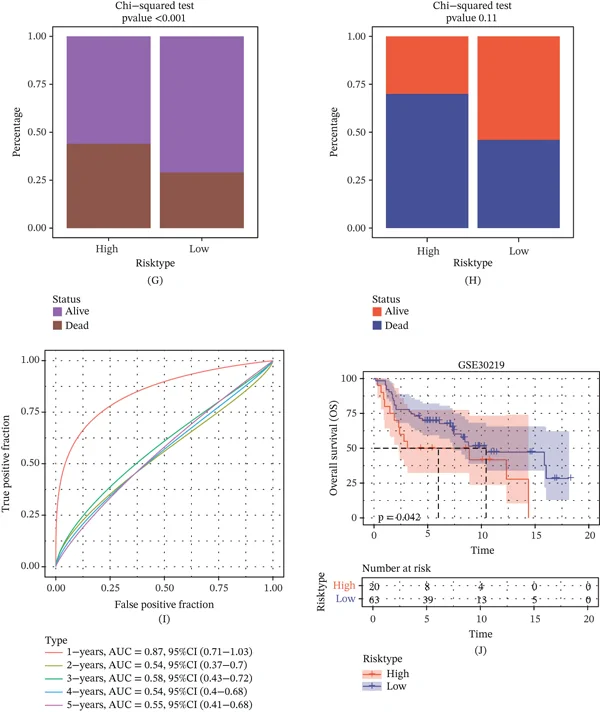
(B)

ROC analysis showed that the model had high AUC values, indicating a high accuracy in distinguishing patients with high and low risk (Figure 4D). KM curve displayed a notable difference in DSS and OS between the two risk groups. Specifically, in the TCGA‐LUAD training cohort, patients with higher RiskScore had a poorer outcome (Figure 4E,F, *p* < 0.001). Analysis of the survival status demonstrated that the high‐risk group had more death cases than the low‐risk group (Figure 4G). The stability and reliability of the model were also validated using the validation dataset GSE30219. As expected, the results in both the training set and validation dataset were consistent (Figure 4H). In addition, the model also reached a high AUC value in the validation set (Figure 4I). Similar to the training set results, patients with high RiskScore showed a poorer outcome, whereas those with low RiskScore exhibited a better prognosis (Figure 4J, *p* < 0.05).

To further validate the robustness and predictive performance of the model, we also conducted external validation using the GSE3141 dataset. The results showed a significantly higher proportion of death cases in the high‐risk group than in the low‐risk group (Supporting Information 1: Figure S1A, *p* < 0.001). ROC curve analysis revealed that the model′s AUC values ranged from 0.58 to 0.63 over 1–5 years (Supporting Information 1: Figure S1B). Survival analysis further demonstrated that the OS rate of the high‐risk patients was significantly lower than that of low‐risk patients (Supporting Information 1: Figure S1C, *p* = 0.001), validating the model′s stability and generalizability in an independent dataset.

Further proteomic analysis revealed that multiple model genes exhibited significantly differential expression at the protein level, with their overall expression trends highly consistent with those observed in transcriptomic analysis. However, the expression differences of several proteins were not statistically significant (Supporting Information 2: Figure S2).

### 3.5. Development of a Nomogram

**Figure 5:**
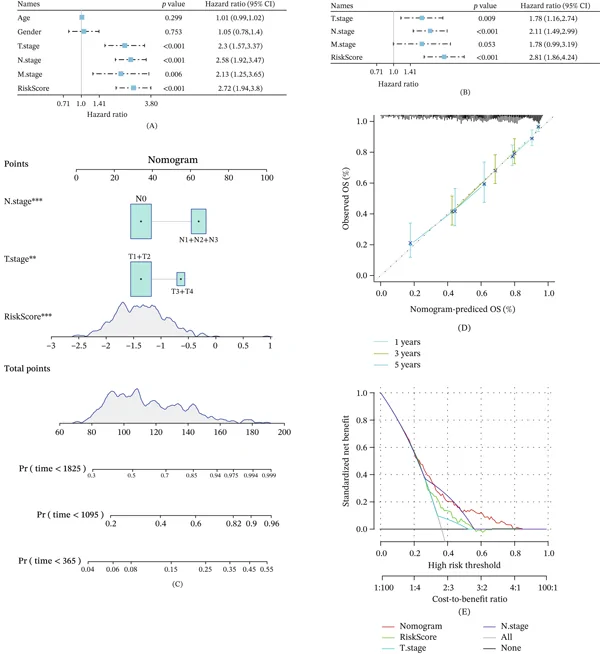
Development of a nomogram integrating RiskScore and clinicopathological factors and validation. (A) Univariate Cox analysis of RiskScore and clinical characteristics. The asterisk significance is defined as ∗∗*p* < 0.01 and ∗∗∗*p* < 0.001. (B) Multivariate Cox analysis of risk score and clinical characteristics. (C) OS prediction by a nomogram that integrated T stage, N stage, and RiskScore. (D) Calibration plots showing strong agreement between 1‐, 3‐, and 5‐year actual and predicted survival. (E) DCA illustrating higher net clinical benefit from the nomogram than individual indicators.

Univariate and multivariate Cox regression analyses demonstrated that T stage, N stage, and RiskScore were all independent prognostic factors for the OS of LUAD patients (Figure 5A,B). Subsequently, we developed a nomogram prediction model incorporating T stage, N stage, and RiskScore (Figure 5C). The calibration curve demonstrated that the model‐predicted survival rates closely aligned with actual observed values, indicating excellent predictive performance (Figure 5D). Decision curve analysis (DCA) results further confirmed that the nomogram had greater clinical net benefit compared with using T staging, N staging, or RiskScore alone (Figure 5E).

### 3.6. *MYO1F* Transfection and In Vitro Experiments

**Figure 6:**
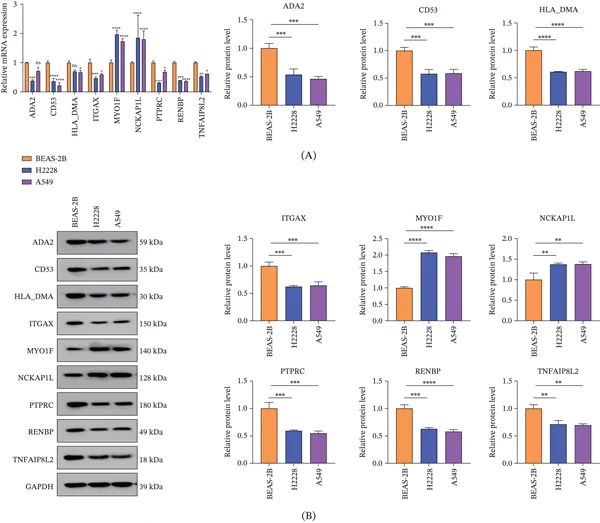
Verification of the expression of nine key genes. (A) Relative mRNA expression of nine prognostic biomarkers in BEAS‐2B, H2228, and A549 measured through qRT‐PCR test (*n* = 3). (B) Relative protein expression of nine prognostic biomarkers in BEAS‐2B, H2228, and A549 measured by WB. ns means not significant (*n* = 3).  ^∗∗∗∗^ *p* < 0.0001,  ^∗∗∗^ *p* < 0.001,  ^∗∗^ *p* < 0.01, and  ^∗^ *p* < 0.05.

The qRT‐PCR experiment showed that compared with human normal bronchial epithelial cell line (BEAS‐2B), LUAD cell lines (H2228 and A549) had higher expressions of *MYO1F* and NCK‐associated protein 1‐like (*NCKAP1L*) and lower expressions of adenosine Deaminase 2 (*ADA2*), cluster of Differentiation 53 (*CD53*), *HLA-DMA*, Integrin subunit alpha X (*ITGAX*), *PTPRC*, TNFAIP8‐like 2 (*TNFAIP8L2*), and renin binding protein (*RENBP*) (Figure 6A, *p* < 0.05). The results of WB were consistent with those of qRT‐PCR (Figure 6B, *p* < 0.05).

**Figure 7:**
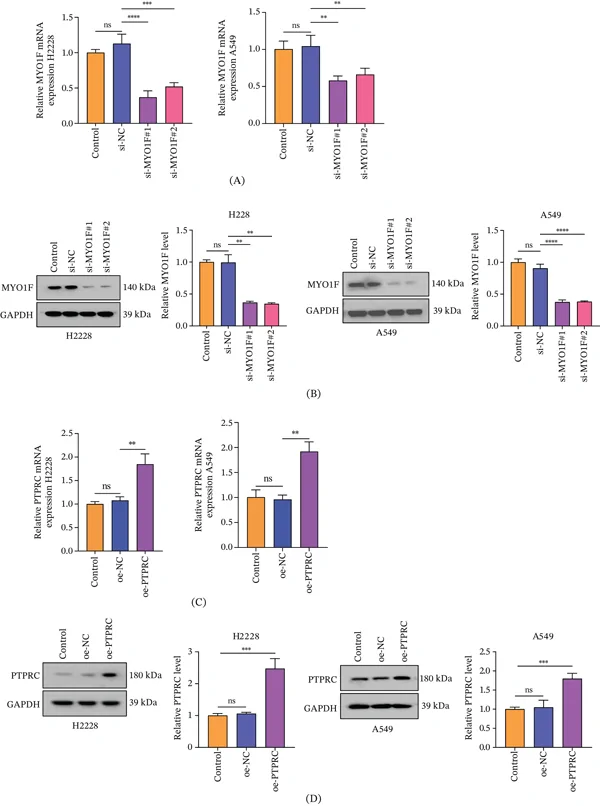
Silencing of *MYO1F* and overexpression of *PTPRC*. (A) The qRT‐PCR verification of the silencing of *MYO1F* in the H2228 and A549 cell lines (*n* = 3). (B) WB verification of the silencing of *MYO1F* in the H2228 and A549 cell lines (*n* = 3). (C) The qRT‐PCR verification of the overexpression of *PTPRC* in the H2228 and A549 cell lines (*n* = 3). (D) WB verification of the overexpression of *PTPRC* in the H2228 and A549 cell lines (*n* = 3). ns means not significant.  ^∗∗∗∗^ *p* < 0.0001,  ^∗∗∗^ *p* < 0.001, and  ^∗∗^ *p* < 0.01.

**Figure 8:**
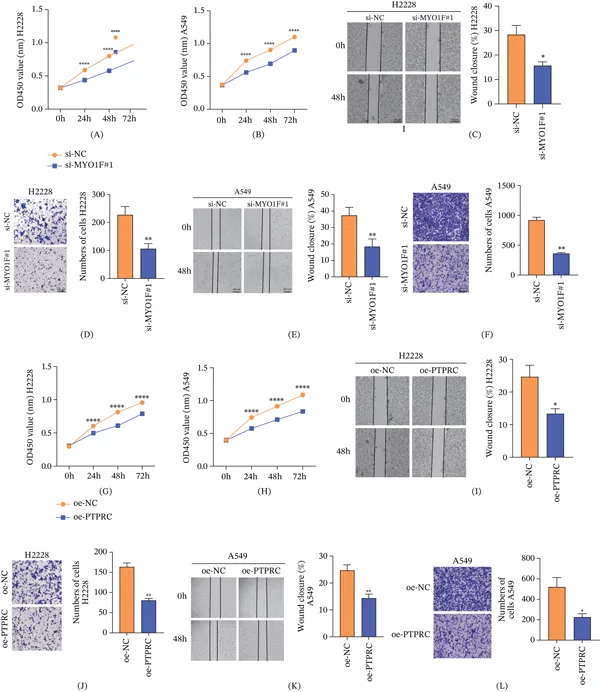
In vitro verification experiments utilizing LUAD cells. (A) Cell viability of H2228 cells after *MYO1F* silencing detected with the CCK‐8 test (*n* = 3). (B) Cell viability of A549 cells after *MYO1F* silencing measured by the CCK‐8 test (*n* = 3). (C, D) Migration and invasion capabilities of H2228 cells after *MYO1F* silencing measured by wound healing and Transwell experiments (*n* = 3). (E, F) The migratory and invasive capabilities of A549 cells following the silencing of *MYO1F*, which were assessed through wound healing and Transwell experiments (*n* = 3). (G) Cell viability of H2228 cells after *PTPRC* overexpress detected with the CCK‐8 test (*n* = 3). (H) Cell viability of A549 cells after *PTPRC* overexpress measured by the CCK‐8 test (*n* = 3). (I, J) Migration and invasion capabilities of H2228 cells after *PTPRC* overexpress measured by wound healing and Transwell experiments (*n* = 3). (K, L) The migratory and invasive capabilities of A549 cells following *PTPRC* overexpress, which were assessed through wound healing and Transwell experiments (*n* = 3). ns means not significant.  ^∗∗∗∗^ *p* < 0.0001,  ^∗∗^ *p* < 0.01, and ∗*p* < 0.05.

*MYO1F* and *PTPRC* were identified as the genes with the highest and lowest expression levels, respectively. Thus, these two genes were selected for further validation. The qRT‐PCR and WB assays confirmed successful knockdown of *MYO1F* and overexpression of *PTPRC* (Figure 7A–D, *p* < 0.05). Subsequently, CCK‐8, wound healing, and Transwell assays showed that the proliferation, migration, and invasion abilities of LUAD cells (H2228 and A549) were markedly inhibited following MYO1F knockdown (Figure 8A–L, *p* < 0.05). Similarly, *PTPRC* overexpression also significantly suppressed the malignant phenotypes of LUAD cells (Figure 8A–L, *p* < 0.05).

### 3.7. Correlation Analysis Between the RiskScore and Immune Function

**Figure 9:**
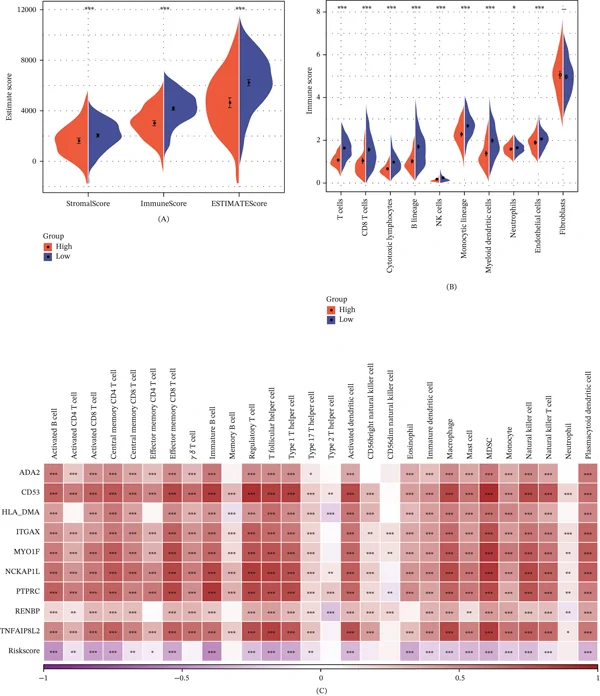
Correlation between immune function and RiskScore. (A) The cell infiltration score by ESTIMATE. (B) The immune infiltration evaluated by MCPcounter. (C) The correlations among the immune infiltration scores evaluated by ssGSEA, RiskScore, and the prognosis genes.  ^∗^ *p* < 0.05,  ^∗∗^ *p* < 0.01, and  ^∗∗∗^ *p* < 0.001.

The estimate scores of the TCGA dataset across different groups were calculated by the ESTIMATE algorithm. The results conspicuously demonstrated that the stromal score, immune score, and estimate score were significantly higher in the low‐risk group (Figure 9A, *p* < 0.001). Furthermore, the MCPcounter algorithm revealed that the infiltration scores of T cells, monocytic lineage cells, neutrophils, myeloid dendritic cells, B lineage cells, NK cells, endothelial cells, CD8 T cells, and cytotoxic lymphocytes were lower in the high‐risk group. On the contrary, these fibroblast infiltration scores were higher in the high‐risk group (Figure 9B, *p* < 0.05). The ssGSEA showed that immature dendritic cells, Type 1 T helper cells, macrophages, activated B cells, T follicular helper cells, immature B cells, regulatory T cells, Type 17 T helper cells, activated CD8 T cells, central memory CD4 T cells, mast cells, MDSCs, monocytes, plasmacytoid dendritic cells, natural killer T cells, natural killer cells, central memory CD8 T cells, effector memory CD8 T cells, and activated dendritic cells were positively correlated with the nine biomarkers and negatively associated with the RiskScore (Figure 9C, *p* < 0.05).

### 3.8. Drug Sensitivity Analysis

**Figure 10:**
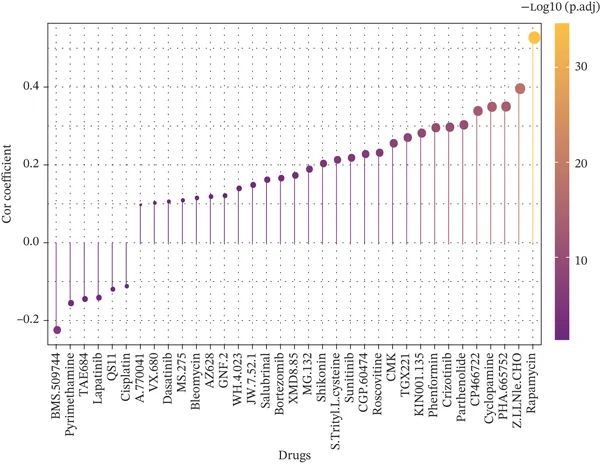
The relationship between drug IC_50_ and RiskScore in the TCGA‐LUAD cohort.

Correlation analysis between the drug IC_50_ and RiskScore revealed that the IC_50_ of the majority of drugs, for instance, rapamycin, Z‐LLNle‐CHO, PHA‐665752, cyclopamine, crizotinib, CP466722, parthenolide, and phenformin, was positively correlated with the RiskScore. This suggests that LUAD patients with a high RiskScore may have lower sensitivity to these drugs, but those with a low RiskScore may be more suitable for taking these medications (Figure 10). Conversely, the IC_50_ of drugs such as pyrimethamine, lapatinib, and cisplatin was negatively correlated with the RiskScore, suggesting that these agents might be candidate drugs for patients in the high‐risk group (Figure 10).

## 4. Discussion

LUAD continues to exhibit high incidence and mortality rates worldwide [32]. PD‐L1/PD‐1 serves as a key immunotherapeutic target in LUAD [33]. Previous studies have linked PD‐L1 expression to poor clinical outcomes [34]. However, the comprehensive expression profile of PD‐1 and its related prognostic biomarkers in LUAD remains poorly understood. In this study, we identified nine biomarkers and established a prognostic model with high reliability. We further explored the potential function of PD‐1 in the immune evasion of LUAD cells and uncovered new biomarkers, thus offering novel insights into the pathogenesis of LUAD. The established prognostic model may serve as a practical tool for assessing patient prognosis and guiding personalized treatment strategies.

We discovered nine prognostic biomarkers, namely, *ADA2*, *CD53*, human leukocyte antigen–DM alpha chain (*HLA-DMA*), *ITGAX*, *MYO1F*, *NCKAP1L*, *PTPRC*, *RENBP*, and *TNFAIP8L2*, with distinct immunological and molecular functions in LUAD. *ADA2* is a purine catabolic enzyme that catalyzes the deamination of adenosine [35]. *ADA2* deficiency impairs M2 macrophage differentiation and exhibits autocrine activity during monocyte proliferation [36]. Reduced ADA activity is associated with advanced LUAD progression [37]. *CD53* is a tetraspanin that regulates lymphocyte recirculation and immune responses [38]. Its expression is predictive of NSCLC patients′ responsiveness to immunotherapy [39] and may serve as a promising biomarker for LUAD [40]. Through major histocompatibility complex (MHC) Class II molecules, *HLA-DMA* regulates antigen presentation [41]. Downregulated *HLA-DMA* is linked to poor prognosis in LUAD [42] and impaired activation of Th1 CD4^+^ T cells [41]. *ITGAX* is a transmembrane glycoprotein that marks immune cells (e.g., macrophages and dendritic cells) [43] and acts as a receptor for the extracellular matrix (ECM) [44]. It has been confirmed as a diagnostic biomarker for LUAD [45]. Unconventional *MYO1F* [46] is crucial for the transmigration and three‐dimensional motility of neutrophils and may regulate cancer cell adhesion [47] and tumor‐associated immune responses [48]. Our present study discovered that silencing *MYO1F* weakened the viability, migration, and invasion of LUAD cells. *NCKAP1L* regulates lymphocyte development and macrophage phagocytosis [49]. Previous studies have confirmed that *NCKAP1L* may influence cancer development by affecting macrophage phagocytosis and antigen presentation [50]. *PTPRC* functions as a pan‐leukocyte marker which regulates antigen receptor signaling [51]. It interacts with PD‐L1 and influences immune infiltration and immunotherapy efficacy in LUAD [52]. *RENBP* is a regulator of the renin–angiotensin system [53], and its overexpression is correlated with a favorable prognosis in male LUAD [54]. *TNFAIP8L2* and *TIPE2* are immunoregulatory factors that maintain immune homeostasis [55]. Members of the *TNFAIP8* family are downregulated in LUAD tissues compared with adjacent normal tissues [56]. Collectively, these biomarkers modulate the tumor immune microenvironment to promote LUAD progression, highlighting their potential as therapeutic targets and predictive biomarkers.

The results of multiple immune infiltration algorithms consistently demonstrated that the low‐risk group exhibited a more active immune microenvironment, characterized by higher stromal and immune scores and increased infiltration of key immune effector cells such as CD8^+^ T cells. As central components of the immune system, these cells maintain tissue homeostasis and eliminate transformed or damaged cells, thereby providing essential antitumor defense [57]. During tumor development, malignant cells dynamically interact with immune cells, shaping the tumor microenvironment toward either an immune‐activated or immunosuppressive state [58]. The low‐risk group identified by our model displayed features of a hot immune phenotype, marked by active cytotoxic immune responses and robust antigen presentation. In contrast, the high‐risk group was associated with immune exhaustion, reflecting a cold or immunosuppressive phenotype. Collectively, these findings suggest that the RiskScore not only predicts clinical outcomes but also captures the functional status of the immune landscape, providing mechanistic insight into tumor–immune interactions and potential implications for immunotherapy responsiveness.

Additionally, this study revealed marked differences in the adaptability of drug treatment between the two risk groups. Low‐risk patients showed greater sensitivity to rapamycin and crizotinib, whereas the high‐risk group was more sensitive to pyrimethamine and cisplatin. Rapamycin, a mammalian target of rapamycin (mTOR) inhibitor, exhibits strong tumor‐suppressive efficacy in cancer treatment [59]. In the low‐risk tumor microenvironment, where immunosuppression is relatively mild, rapamycin may synergistically enhance T‐cell function, reverse immune exhaustion, and delay tumor recurrence [60]. Crizotinib, a tyrosine kinase inhibitor (TKI) used to treat advanced NSCLC patients with ALK positivity [61], may disrupt PD‐L1/PD‐1‐mediated immune escape and enhance CD8^+^ T‐cell activity, thereby bolstering antitumor immune surveillance [60]. In contrast, pyrimethamine is an antiparasitic drug originally used for the treatment of malaria and toxoplasmosis [62]. Pyrimethamine has been shown to selectively inhibit dihydrofolate reductase (DHFR) in LUAD cells, interfering with folate metabolism and offering a potential therapeutic option for lung cancer [63]. Cisplatin induces reactive oxygen species through lipid peroxidation, activating p53 signaling and cell cycle arrest [64], mechanisms that support its clinical application in LUAD [65]. In conclusion, these agents represent promising candidates for personalized treatment strategies in LUAD patients with different risk stratifications, though further studies are needed to validate and optimize their clinical application.

Although the LUAD prognostic risk model constructed in this study exhibited favorable predictive performance, several limitations still need to be addressed. First, all analyses were performed based on public databases and lacked validation in clinical samples. The absence of protein‐level evidence from human tissues may limit the clinical translational value of this research. Future studies should collect paired tumor and adjacent normal tissues from LUAD patients and employ IHC and multiplex immunofluorescence staining to verify the protein expression and tissue localization of core model genes, thereby enhancing clinical translational potential. Second, there are inconsistent descriptions of computational analyses. Specifically, multivariate Cox regression was used to adjust for clinical confounders and identify independent prognostic factors in the results section, but the corresponding rationale was not adequately addressed, weakening the overall logical coherence. Subsequent revisions will ensure consistency across the methods, results, and discussion sections. Third, drug sensitivity predictions solely relied on computational analyses such as pRRophetic and cell line–based in silico analysis, without experimental or real‐world clinical validation. Therefore, definitive conclusions regarding clinical drug applicability, particularly for targeted agents (e.g., crizotinib) that specifically act on gene alterations, cannot be drawn. Further in vitro cell experiments will be performed to examine changes in PD‐L1 expression after knockdown of key model genes, with or without IFN‐*γ* stimulation. Flow cytometry and WB assays will be used to clarify the underlying regulatory mechanisms and provide solid experimental support for drug screening and molecular research.

## 5. Conclusion

In this study, nine biomarkers were successfully identified to develop a prognostic model for LUAD, which provides a novel perspective for predicting patient prognosis. The differential drug sensitivity observed between risk groups may guide precise medication selection, thereby improving therapeutic efficacy and reducing unnecessary adverse effects. However, its specific biological functions require further experimental verification. In‐depth exploration of the characteristics of the TIME may reveal potential targets for the development of novel immunotherapeutic approaches, ultimately contributing to improved treatment outcomes and survival in LUAD.

**Nomenclature**
ADA2: adenosine Deaminase 2
ALK: anaplastic lymphoma kinase
AUC: area under the curve
BP: biological process
CC: cellular component
CCK‐8: Cell Counting Kit‐8
CD53: cluster of Differentiation 53
DEG: differentially expressed gene
DHFR: human dihydrofolate reductase
DSS: disease‐specific survival
ECM: extracellular matrix
FBS: fetal bovine serum
FDR: false discovery rate
FPKM: fragments per kilobase of exon per million reads mapped
GEO: Gene Expression Omnibus
GO: Gene Ontology
HLA‐DMA: human leukocyte antigen–DM alpha chain
IC_50_
: half‐maximal inhibitory concentration
ICI: immune checkpoint inhibitor
IHC: immunohistochemical
ITGAX: integrin subunit alpha X
KEGG: Kyoto Encyclopedia of Genes and Genomes
KM: Kaplan–Meier
LUAD: lung adenocarcinoma
MF: molecular function
MHC: major histocompatibility complex
mTOR: mammalian target of rapamycin
MYO1F: myosin 1F
NCKAP1L: NCK‐associated protein 1‐like
NSCLC: non–small cell lung cancer
OS: overall survival
PD‐1: programmed cell death Protein 1
PD‐L1: programmed death‐ligand 1
PTPRC: protein tyrosine phosphatase receptor Type C
qRT‐PCR: quantitative reverse transcription PCR
RENBP: renin binding protein
RNA‐seq: RNA sequencing
ROC: receiver operating characteristic
ssGSEA: single‐sample gene set enrichment analysis
TCGA: The Cancer Genome Atlas
TIL: tumor‐infiltrating T lymphocyte
TIME: tumor immune microenvironment
TKI: tyrosine kinase inhibitor
TME: tumor microenvironment
TNFAIP8L2: TNFAIP8‐like 2
WB: western blot
WGCNA: weighted gene coexpression network analysis

## Author Contributions

All authors contributed to the present work: B.J. and T.G. designed the study; C.C. acquired the data; B.S. and Z.Z. interpreted the data; B.J. drafted the manuscript; Z.Z. revised the manuscript. B.J. and T.G. contributed equally to this work.

## Funding

No funding was received for this manuscript.

## Disclosure

All authors read and approved the manuscript.

## Ethics Statement

Ethical approval was not required for this study because it did not involve any human experiments.

## Consent

The authors have nothing to report.

## Conflicts of Interest

The authors declare no conflicts of interest.

## Supporting Information

Additional supporting information can be found online in the Supporting Information section.

## Supporting information


**Supporting Information 1** Figure S1: External validation of the prognostic model using the GSE3141 dataset. (A) Risk scores and survival statuses in the GSE3141 dataset. (B) Time‐dependent ROC curves for 1–5‐year overall survival demonstrating good predictive performance (AUC values of 0.59, 0.61, 0.63, 0.6, and 0.58, respectively). (C) Kaplan–Meier survival analysis revealing that high‐risk patients had significantly worse overall survival than low‐risk patients (*p* = 0.001).

**Supporting Information 2** Figure S2: Protein expression validation of nine prognostic model genes based on CPTAC‐LUAD proteomic data from the UALCAN database. The UALCAN platform (CPTAC‐LUAD) was used to compare the protein expression levels of nine prognostic genes, including HLA‐DMA, ITGAX, MYO1F, NCKAP1L, PTPRC, TNFAIP8L2, RENBP, CD53, and ADA2, between normal lung tissues and primary LUAD tumor tissues. Box plots present the standardized protein expression values (*Z*‐value), and the statistical significance of differences among groups is indicated in each subgraph.

**Supporting Information 3** Table S1: The primary antibody utilized in this study.

**Supporting Information 4** Table S2: Differentially expressed genes between the high‐expression group and the low‐expression group of PDCD1.

**Supporting Information 5** Table S3: Results of univariate Cox regression analysis.

## Data Availability

The datasets generated and/or analyzed during the current study are available in the GSE30219 repository (https://www.ncbi.nlm.nih.gov/geo/query/acc.cgi?acc=GSE30219). The remaining analysis codes and intermediate processed data are available from the corresponding author upon reasonable request.
