# Supplementary material for: Identification of PD‐1‐Related Genes as Prognostic Biomarkers in Lung Adenocarcinoma
Source: Hum Mutat. 2026 Aug 25;2026:8948469. doi: 10.1155/humu/8948469 (PMC13504365; doi:10.1155/humu/8948469)
Supplement: Supplementary file 1 [file HUMU-2026-8948469-s001.pdf]

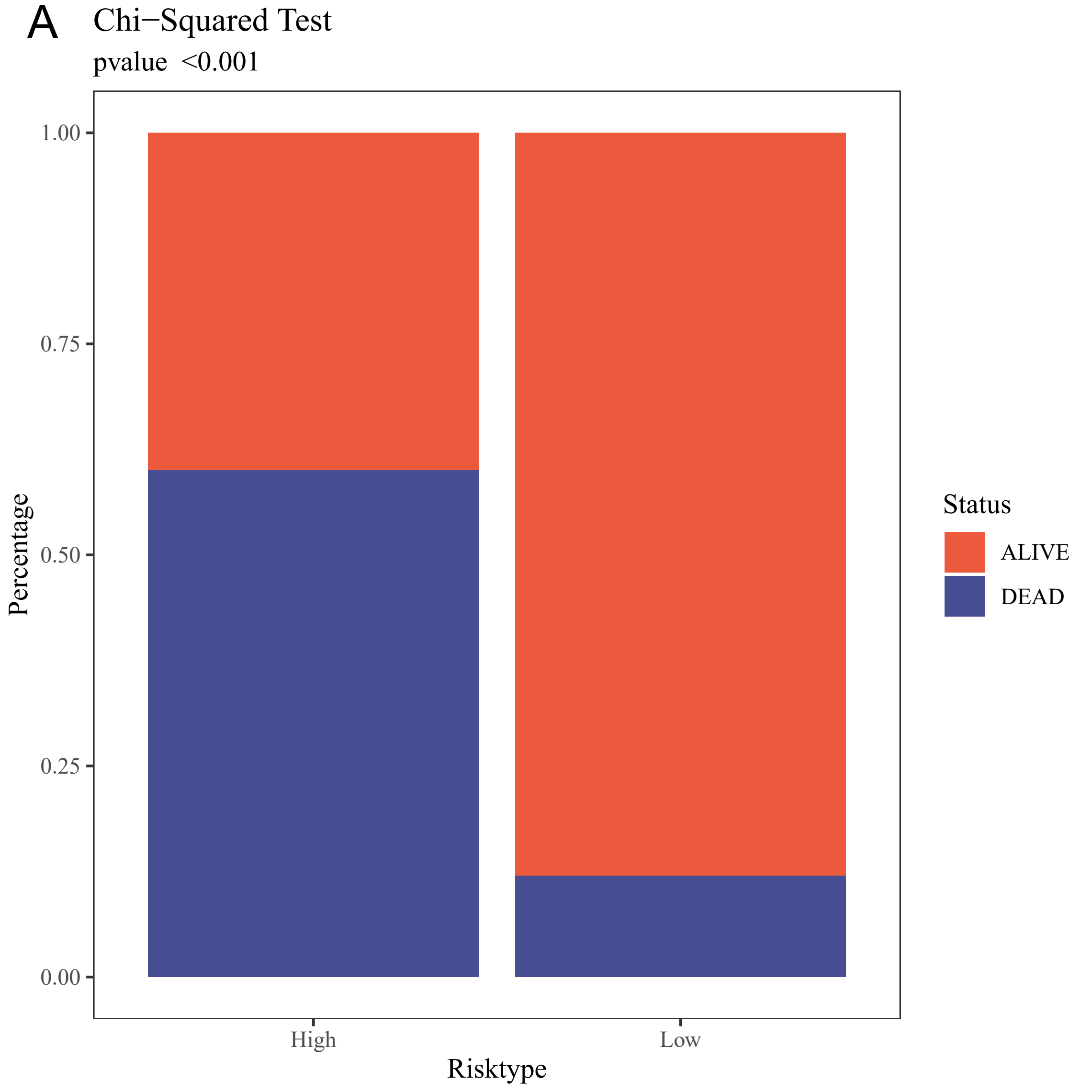
A
Chi−Squared Test
pvalue <0.001
1.00
0.75
0.50
0.25
0.00
Percentage
High
Low
Risktype
Status
ALIVE
DEAD
