# Supplementary material for: Identification of PD‐1‐Related Genes as Prognostic Biomarkers in Lung Adenocarcinoma
Source: Hum Mutat. 2026 Aug 25;2026:8948469. doi: 10.1155/humu/8948469 (PMC13504365; doi:10.1155/humu/8948469)
Supplement: Supplementary file 2 [file HUMU-2026-8948469-s002.pdf]

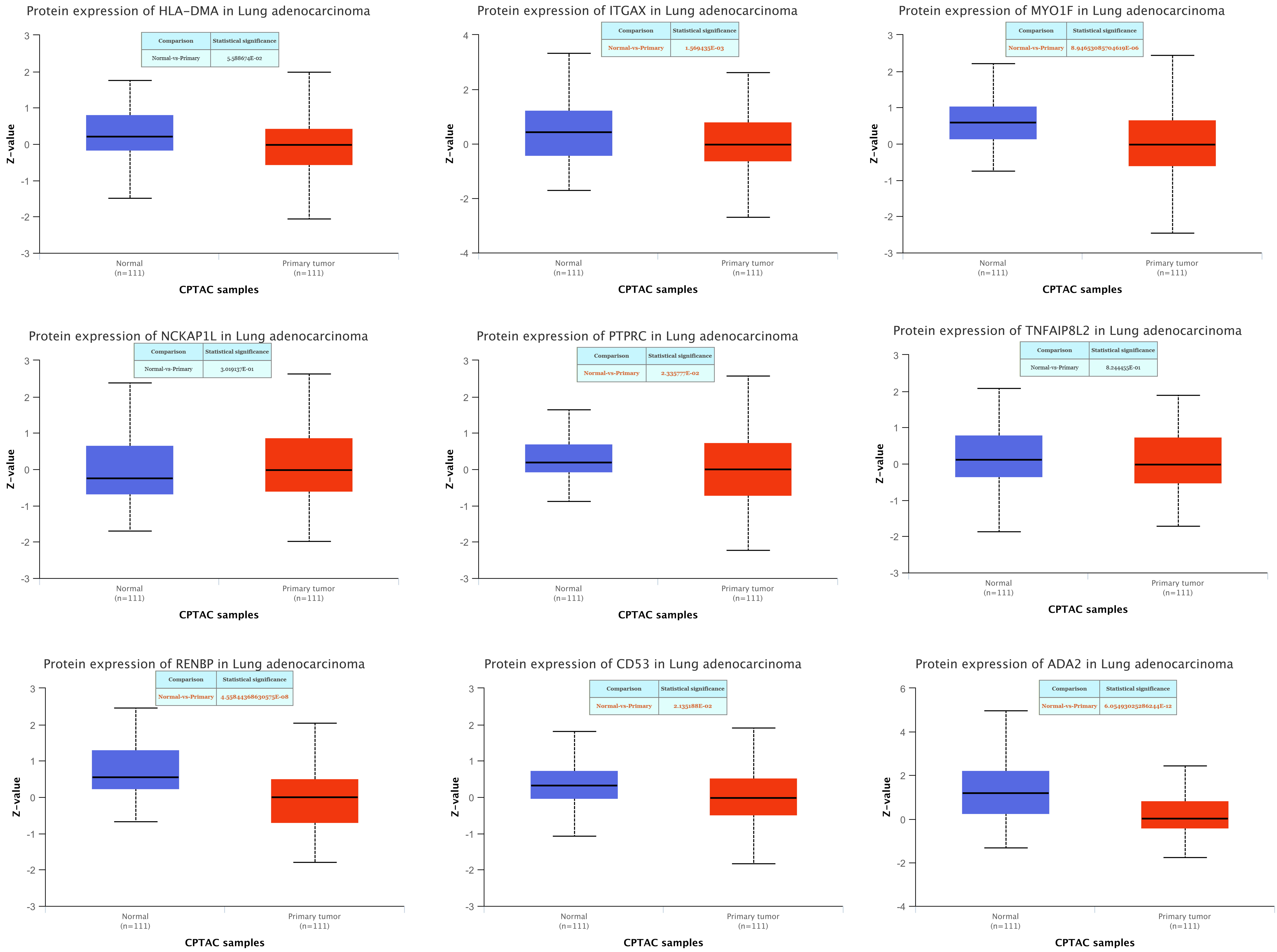

Protein expression of HLA–DMA in Lung adenocarcinoma
Comparison | Statistical significance
Normal-vs-Primary | 5.588674E-02
Z–value
Normal (n=111)
Primary tumor (n=111)
CPTAC samples
Protein expression of ITGAX in Lung adenocarcinoma
Comparison | Statistical significance
Normal-vs-Primary | 1.569435E-03
Z–value
Normal (n=111)
Primary tumor (n=111)
CPTAC samples
Protein expression of MYO1F in Lung adenocarcinoma
Comparison | Statistical significance
Normal-vs-Primary | 8.94653085704619E-06
Z–value
Normal (n=111)
Primary tumor (n=111)
CPTAC samples
Protein expression of NCKAP1L in Lung adenocarcinoma
Comparison | Statistical significance
Normal-vs-Primary | 3.019137E-01
Z–value
Normal (n=111)
Primary tumor (n=111)
CPTAC samples
Protein expression of PTPRC in Lung adenocarcinoma
Comparison | Statistical significance
Normal-vs-Primary | 2.335777E-02
Z–value
Normal (n=111)
Primary tumor (n=111)
CPTAC samples
Protein expression of TNFAIP8L2 in Lung adenocarcinoma
Comparison | Statistical significance
Normal-vs-Primary | 8.244455E-01
Z–value
Normal (n=111)
Primary tumor (n=111)
CPTAC samples
Protein expression of RENBP in Lung adenocarcinoma
Comparison | Statistical significance
Normal-vs-Primary | 4.55844368630575E-08
Z–value
Normal (n=111)
Primary tumor (n=111)
CPTAC samples
Protein expression of CD53 in Lung adenocarcinoma
Comparison | Statistical significance
Normal-vs-Primary | 2.135188E-02
Z–value
Normal (n=111)
Primary tumor (n=111)
CPTAC samples
Protein expression of ADA2 in Lung adenocarcinoma
Comparison | Statistical significance
Normal-vs-Primary | 6.05493025286244E-12
Z–value
Normal (n=111)
Primary tumor (n=111)
CPTAC samples
