# Supplementary material for: Identification of PD‐1‐Related Genes as Prognostic Biomarkers in Lung Adenocarcinoma
Source: Hum Mutat. 2026 Aug 25;2026:8948469. doi: 10.1155/humu/8948469 (PMC13504365; doi:10.1155/humu/8948469)
Supplement: Supplementary file 3 [file HUMU-2026-8948469-s003.docx]

Table S1. The primary antibody utilized in this study.

|  | Cat. | Company | Dilution ratio |
| --- | --- | --- | --- |
| ADA2 | ab288296 | Abcam | 1:1000 |
| CD53 | ab134094 | Abcam | 1:2000 |
| HLA_DMA | #97971 | Cell Signaling Technology | 1:1000 |
| ITGAX | ab52632 | Abcam | 1:1000 |
| MYO1F | #54922 | Cell Signaling Technology | 1:1000 |
| NCKAP1L | PA5-58813 | ThermoFisher | 1:1000 |
| PTPRC | #13917 | Cell Signaling Technology | 1:1000 |
| RENBP | PA5-51387 | ThermoFisher | 1:1000 |
| TNFAIP8L2 | ab316848 | Abcam | 1:1000 |
| GAPDH | ab181092 | Abcam | 1:5000 |
