# Supplementary material for: Identification of PD‐1‐Related Genes as Prognostic Biomarkers in Lung Adenocarcinoma
Source: Hum Mutat. 2026 Aug 25;2026:8948469. doi: 10.1155/humu/8948469 (PMC13504365; doi:10.1155/humu/8948469)
Supplement: Supplementary file 4 [file HUMU-2026-8948469-s005.docx]

|  | p.value | HR | Low 95%CI | High 95%CI |
| --- | --- | --- | --- | --- |
| ADA2 | 0.002201 | 0.800455 | 0.694156 | 0.923032 |
| ARHGAP30 | 0.009366 | 0.794218 | 0.66752 | 0.944964 |
| BIN2 | 0.025897 | 0.806958 | 0.668189 | 0.974547 |
| CD37 | 0.01075 | 0.823658 | 0.70959 | 0.956064 |
| CD52 | 0.01153 | 0.858803 | 0.763141 | 0.966456 |
| CD53 | 0.038283 | 0.871503 | 0.765178 | 0.992603 |
| CD74 | 0.010941 | 0.873436 | 0.786982 | 0.969388 |
| CLEC10A | 0.004493 | 0.795539 | 0.679419 | 0.931506 |
| CSF2RB | 0.009648 | 0.806366 | 0.685096 | 0.949101 |
| DOCK2 | 0.021096 | 0.795464 | 0.654883 | 0.966223 |
| EVI2B | 0.01347 | 0.834649 | 0.723177 | 0.963304 |
| HLA-DMA | 0.000583 | 0.805806 | 0.712522 | 0.911303 |
| HLA-DMB | 0.002148 | 0.823952 | 0.728104 | 0.932419 |
| HLA-DOA | 0.005942 | 0.853536 | 0.762465 | 0.955484 |
| HLA-DPA1 | 0.013129 | 0.878347 | 0.792774 | 0.973156 |
| HLA-DPB1 | 0.005726 | 0.861143 | 0.774499 | 0.957479 |
| HLA-DQA1 | 0.010458 | 0.875354 | 0.790541 | 0.969266 |
| HLA-DQB1 | 0.009182 | 0.882969 | 0.804042 | 0.969643 |
| HLA-DRA | 0.007201 | 0.869263 | 0.784825 | 0.962785 |
| HLA-DRB1 | 0.012987 | 0.881814 | 0.79851 | 0.97381 |
| HLA-DRB5 | 0.005143 | 0.887333 | 0.816058 | 0.964834 |
| IGSF6 | 0.009963 | 0.823903 | 0.711046 | 0.954673 |
| IL10RA | 0.019078 | 0.822108 | 0.697904 | 0.968416 |
| IRF8 | 0.003552 | 0.78608 | 0.668639 | 0.92415 |
| ITGAX | 0.037754 | 0.849138 | 0.727738 | 0.99079 |
| LPXN | 0.013331 | 0.781817 | 0.643352 | 0.950085 |
| MPEG1 | 0.016546 | 0.842024 | 0.731569 | 0.969156 |
| MYO1F | 0.02995 | 0.801331 | 0.65609 | 0.978723 |
| NCKAP1L | 0.027343 | 0.830201 | 0.70372 | 0.979416 |
| PLEK | 0.037883 | 0.868477 | 0.760226 | 0.992142 |
| PTPRC | 0.006269 | 0.830787 | 0.727377 | 0.948898 |
| RENBP | 0.032767 | 0.844557 | 0.723221 | 0.98625 |
| SASH3 | 0.006563 | 0.81384 | 0.701509 | 0.944157 |
| TNFAIP8L2 | 0.028603 | 0.834877 | 0.710302 | 0.981301 |

Table S3. Results of univariate COX regression analysis.
